# An Ab Initio Study of Magnetism in Disordered Fe-Al Alloys with Thermal Antiphase Boundaries

**DOI:** 10.3390/nano10010044

**Published:** 2019-12-23

**Authors:** Martin Friák, Miroslav Golian, David Holec, Nikola Koutná, Mojmír Šob

**Affiliations:** 1 Institute of Physics of Materials, v.v.i., Czech Academy of Sciences, Žižkova 22, CZ-616 62 Brno, Czech Republic; 473873@mail.muni.cz (M.G.); mojmir@ipm.cz (M.Š.); 2 Department of Materials Science, Montanuniversität Leoben, Franz-Josef-Strasse 18, A-8700 Leoben, Austria; david.holec@unileoben.ac.at; 3 Institute of Materials Science and Technology, TU Wien, Getreidemarkt 9, A-1060 Vienna, Austria; nikola.koutna@mail.muni.cz

**Keywords:** Fe-Al, antiphase boundaries, magnetism, ab initio, stability, disorder

## Abstract

We have performed a quantum-mechanical study of a B2 phase of Fe${}_{70}$Al${}_{30}$ alloy with and without antiphase boundaries (APBs) with the {001} crystallographic orientation of APB interfaces. We used a supercell approach with the atoms distributed according to the special quasi-random structure (SQS) concept. Our study was motivated by experimental findings by Murakami et al. (Nature Comm. 5 (2014) 4133) who reported significantly higher magnetic flux density from A2-phase interlayers at the thermally-induced APBs in Fe${}_{70}$Al${}_{30}$ and suggested that the ferromagnetism is stabilized by the disorder in the A2 phase. Our computational study of sharp APBs (without any A2-phase interlayer) indicates that they have moderate APB energies (≈0.1 J/m${}^{2}$) and cannot explain the experimentally detected increase in the ferromagnetism because they often induce a ferro-to-ferrimagnetic transition. When studying thermal APBs, we introduce a few atomic layers of A2 phase of Fe${}_{70}$Al${}_{30}$ into the interface of sharp APBs. The averaged computed magnetic moment of Fe atoms in the whole B2/A2 nanocomposite is then increased by 11.5% w.r.t. the B2 phase. The A2 phase itself (treated separately as a bulk) has the total magnetic moment even higher, by 17.5%, and this increase also applies if the A2 phase at APBs is sufficiently thick (the experimental value is 2–3 nm). We link the changes in the magnetism to the facts that (i) the Al atoms in the first nearest neighbor (1NN) shell of Fe atoms nonlinearly reduce their magnetic moments and (ii) there are on average less Al atoms in the 1NN shell of Fe atoms in the A2 phase. These effects synergically combine with the influence of APBs which provide local atomic configurations not existing in an APB-free bulk. The identified mechanism of increasing the magnetic properties by introducing APBs with disordered phases can be used as a designing principle when developing new magnetic materials.

## 1. Introduction

Antiphase boundaries (APBs) are very frequently occurring extended defects in crystals containing ordered sublattices. They are at the interface of two regions of the same ordered phase which are shifted one with respect to the other. The shift is formed, for example, during ordering processes when two grains crystallizing from the melt have the origin of their lattices in a distance which is not a multiple of translational vectors of the superlattice. As the formation of the above described interfaces occurs at elevated temperatures when diffusion processes are sufficiently active, an intermediate disordered phase can form (so-called thermal APBs). Dislocations with Burgers vectors that are not translation vectors of the ordered superlattice can also create APBs at any temperature, as they move through an ordered phase (so-called deformation APBs with sharp interfaces).

Our theoretical study is focused on APBs in Fe${}_{70}$Al${}_{30}$. This alloy belongs into a very promising family of Fe-Al-based materials possessing interesting properties including, e.g., remarkable resistance to oxidation, relatively low density, electrical resistivity, or low cost of raw materials [1,2,3,4,5,6,7,8]. Consequently, they have been very intensively studied both experimentally (see, e.g., Refs. [9,10,11,12,13,14,15,16,17,18,19]) and theoretically (see, e.g., Refs. [20,21,22,23,24,25,26,27,28,29,30,31,32,33,34,35,36,37,38,39,40,41]). Focusing on APBs, they have been observed in Fe-Al-based materials with sublattices by means of the transmission electron microscopy (TEM). For example, Marcinkowski and Brown in their classical works [42,43] observed APBs in thin foils of Fe-Al alloys by TEM and reported two types of APBs for the D0${}_{3}$ superlattice. One of them is specific to the D0${}_{3}$ superlattice but the other one, which is crucial for our study, can appear also in the B2 lattice. It is characterized by a shift of the interfacing grains by the 1/2〈111〉*a* where *a* is the lattice parameter of the 2-atom elementary cell of the body-centered cubic (bcc) lattice. It interrupts the chemical order of the first nearest neighbors (APB-NN or APB-B2 type). As deformation APBs, both types separate partials of superdislocations in Fe-Al materials [44,45,46]. Other studies may be found in Refs. [47,48,49,50,51,52].

Our research is focused on APB-NN (APB-B2) in a B2-phase Fe${}_{70}$Al${}_{30}$, and it was motivated by recent experiments by Murakami et al. [50]. Murakami and co-workers combined TEM characterization with direct magnetization measurements of thermally induced APBs. They were found to possess a finite width (2–3 nm) and a significant atomic disorder (an A2 phase). Importantly, electron holography studies of Murakami et al. revealed a magnetic flux density at the APBs higher than that of the matrix by approximately 60% (at 293 K). The authors concluded that the ferromagnetic state of APBs is stabilized by the disorder within APBs. To test this interpretation, we performed a theoretical study of APBs in the B2-phase of Fe${}_{70}$Al${}_{30}$. We first simulated sharp APBs and, after identifying important structure-property relations, we expanded the sharp interfaces of APBs by interlayers of disordered A2 phase. The Fe atoms in the A2 phase indeed exhibit (on average) a higher magnetic moment.

## 2. Methods

**Figure 1:**
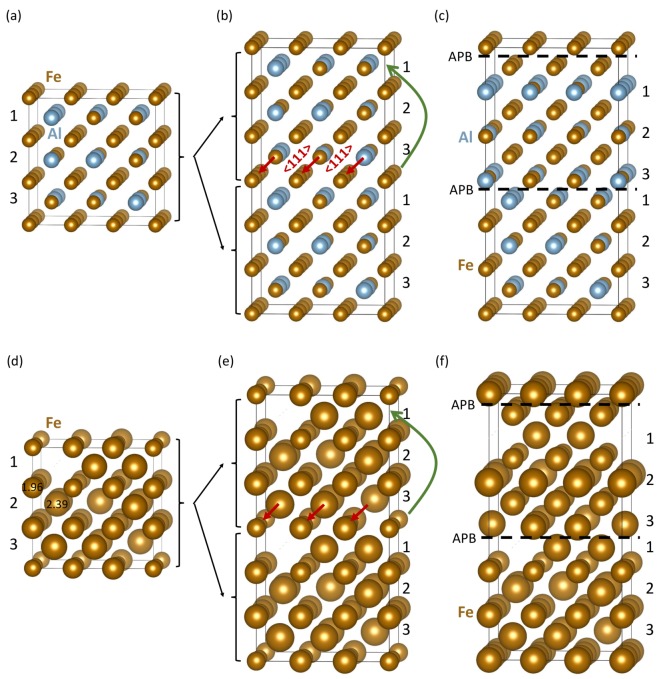
Schematic visualizations of our way of constructing sharp antiphase boundaries (APBs) in Fe${}_{70}$Al${}_{30}$ alloy. A 54-atom supercell in part (**a**) is a 3 × 3 × 3 multiple of 2-atom body-centered cubic (bcc) elementary cell and it is our model of the B2 phase of Fe${}_{70}$Al${}_{30}$. It consists of atomic planes (i) containing only Fe atoms which are separated by planes (ii) containing both Fe and Al atoms (the latter with the atoms distributed according to the special quasi-random structure (SQS) concept). As each Fe-Al plane is different, they are numbered 1–3. Two of these 54-atom cells stacked along the [001] direction form a supercell (**b**) which was used for constructing the studied APBs. In particular, when applying a 〈111〉 shift to all atoms in the upper half of the supercell (**b**), a supercell (**c**) with two sharp APBs (see dashed lines) is formed. The 〈111〉 shift is indicated by the red vectors in part (**b**). In order to apply the 〈111〉 shift to all atoms in the upper half of (**b**), one Fe atomic plane is cyclically shifted as schematically visualized by a curved green arrow. The computed local magnetic moments of atoms in supercells (**a**–**c**) are shown in parts (**d**–**f**), respectively. The magnitude of local magnetic moments are visualized by the diameter of spheres representing atoms—two values in Bohr magnetons $\mu_{B}$ are given in (**d**) in order to show the scaling.

Our quantum-mechanical calculations were done with the help of the Vienna Ab initio Simulation Package (VASP) [53,54]. The software implements the density functional theory [55,56]. We have utilized projector augmented wave (PAW) pseudopotentials [57,58]. The generalized gradient approximation (GGA) for the exchange and correlation energy was employed in the parametrization according to Perdew and Wang [59] (PW91) in combination with the Vosko–Wilk–Nusair correction [60]. The plane-wave energy cut-off was equal to 400 eV and the product of the number of Monkhorst–Pack k-points and the number of atoms was equal to 27 648 (e.g., 8 × 8 × 8 k-point mesh in the case of 54-atom supercell of the B2 phase in Figure 1a). All studied supercells were fully relaxed (with respect to their atomic positions, cell shape as well as the supercell volume) and the forces were reduced under 0.001 eV/Å. All calculated states were initially set up as ferromagnetic.

The B2 phase of Fe${}_{70}$Al${}_{30}$ is modeled by the special quasi-random structure (SQS) concept [61] and generated by the USPEX code [62,63,64] (see it in Figure 1a). It consists of two types of {001} atomic planes. One contains only Fe atoms and the other one both Fe and Al atoms distributed according to the above-mentioned SQS concept. As each Fe-Al plane has a different distribution of Fe and Al atoms, they are numbered 1–3 in Figure 1a. It is convenient to write the overall composition Fe${}_{70}$Al${}_{30}$ as Fe${}_{50}$(Fe${}_{20}$Al${}_{30}$) distinguishing between the two sublattices (one occupied by solely Fe atoms and the other by both Fe and Al). It is worth mentioning that the Fe-Al sublattice is Al-rich while the overall alloy is Fe-rich (this aspect will be important for our analysis below). As the APB energy typically depends on the crystallographic orientation of the interface only very weakly, we believe that our choice of the {001} interface plane is sufficiently representative for a broader range of orientations.

Two of the B2-phase 54-atom cells shown in Figure 1a stacked along the [001] direction form an 108-atom supercell (Figure 1b) which was used for constructing the studied APBs. We applied a 〈111〉 shift to all atoms in the upper half of the supercell in Figure 1b to form a supercell with two sharp APBs (dashed lines in Figure 1c). Other three atomic configurations of the B2 phase with sharp APBs and our model of the disordered A2 phase at the thermal APBs in Fe${}_{70}$Al${}_{30}$ are described below.

## 3. Results

**Figure 2:**
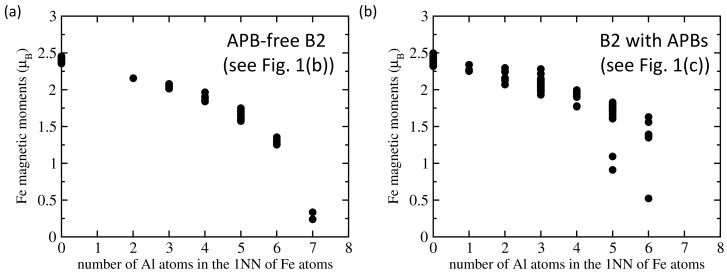
Computed local magnetic moments of Fe atoms as a function of the number of Al atoms in the first nearest neighbor (1NN) shell of Fe atoms. Part (**a**) shows them in the APB-free B2 phase (visualized in Figure 1b) and part (**b**) contains the values for the B2 phase with APBs shown in Figure 1c.

Our results related to both APB-free B2 phase of Fe${}_{70}$Al${}_{30}$ and that containing sharp APBs are given in Figure 1 and Table 1. The APB-free B2 phase is disordered and, therefore, each Fe atom has a different local atomic environment, and these differences are sensitively reflected by the value of their local magnetic moment. The computed magnitude of local magnetic moments in both APB-free B2 phase (Figure 1b) and the APB-containing one (Figure 1c) are visualized in Figure 1e,f, respectively, by spheres with different radius. As the local magnetic moments of Fe atoms in Fe-rich Fe-Al binaries decrease with an increasing number of Al atoms in the first nearest neighbors shell (1NN) of Fe atoms [22,39], we analyze these relations also here. The computed values of local magnetic moments of Fe atoms are plotted as functions of the number of Al atoms in the 1NN in Figure 2. The visualized trends show the local magnetic moments of Fe atoms decreasing with the increasing number of Al atoms in the 1NN of Fe atoms in a qualitative agreement with our previous studies [22,39]. Regarding the Al atoms in the 2NN shell, we did not find any clear impact—see Appendix A.

A statistical summary of the number of Al atoms in the 1NN shell of Fe atoms is given in Table 1. It contains percentages of Fe atoms with different numbers of Al atoms in their 1NN shells. In the APB-free B2 phase, the Fe atoms at the Fe-only sublattice have the atoms at the Fe-Al sublattice as their 1NN neighbors and vice versa. In contrast, the studied APBs introduce two types of APB-specific environments. In particular, one APB is characterized by two adjacent Fe-only atomic planes (see it close to the top of the supercell in Figure 1c). The other one has an interface formed by two adjacent Fe-Al planes containing both Fe and Al atoms (see it in the center of the supercell in Figure 1c).

When comparing the percentages of Fe atoms with different numbers of Al atoms in the APB-free B2 phase and that with the APBs, it is important to realize that the atoms are, due to the presence of APBs, re-distributed so that there are more Fe atoms with fewer Al atoms in the 1NN shells of these Fe atoms. Despite the fact that the number of Fe atoms with no Al atom in the 1NN shell is lower in the case of APB-containing B2 phase, the percentages of Fe atoms with 1, 2, and 3 Al atoms in the 1NN shell are significantly higher in the case of APBs and percentages of Fe atoms with 4 or more Al atoms in the 1NN shell are significantly lower. These two findings result in higher local magnetic moments of the Fe atoms in the APB atomic configurations in Figure 1c. This enhancement of local magnetic moments is also reflected by higher values of the average magnetic moment of Fe atoms listed in Table 1 (an increase from 1.83 to 2.00 $\mu_{B}$). As far as different volumes per atom are concerned (see Table 1), they indicate a possibility of strains which can lead to incoherent APB interfaces, but such states are beyond the scope of our study. Lastly, the APB energy is equal to 0.103 J/m${}^{2}$.

### 3.1. Compositional Changes at Sharp APB Interfaces

**Figure 3:**
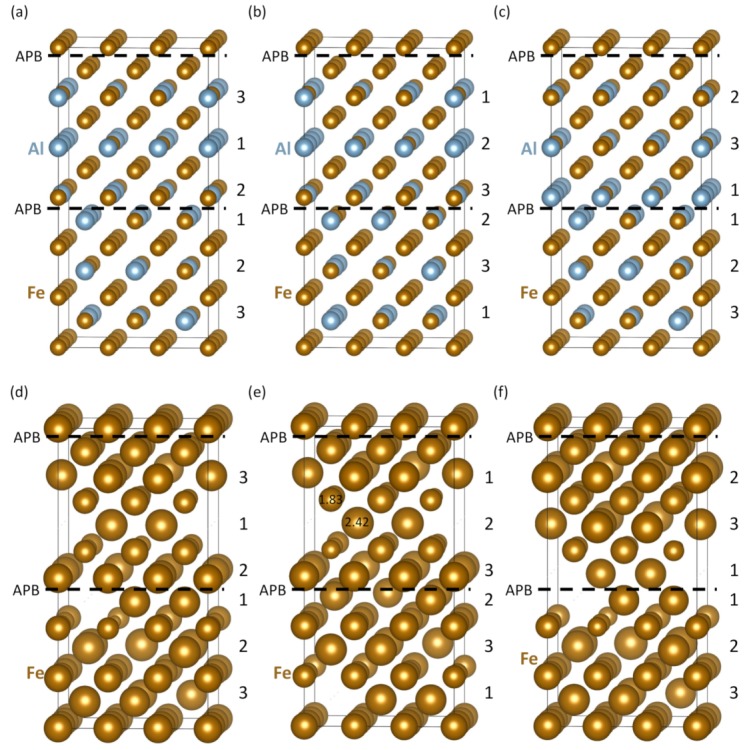
Schematic visualizations of three additional variants of sharp APBs with different compositions of two atomic planes containing both Fe and Al atoms. In particular, two Fe-Al planes above and below the APB in the middle of the supercell in Figure 1c contain 5 and 7 Al atoms, respectively, while those shown here contain 4 and 7 Al atoms in part (**a**), 4 and 4 Al atoms in part (**b**), and 7 and 7 Al atoms in part (**c**). The local magnetic moments of atoms corresponding to supercells (**a**–**c**) are shown in parts (**d**–**f**), respectively. The magnitude of local magnetic moments are visualized by the diameter of spheres representing atoms—two values in Bohr magnetons $\mu_{B}$ are given in part (**e**) in order to show the scaling.

Next, we check the sensitivity of our computed properties of sharp APBs to compositional changes at the APB interfaces. Using the supercell of the B2 phase (Figure 1a) we have applied three different cyclic shifts to the atomic planes in the two interfacing grains in Figure 1c—see these atomic configurations and their corresponding local magnetic moments in Figure 3. As the cyclic shifts were applied to the same supercell of the B2 phase (Figure 1a), we can use its energy as the same reference as before when analyzing the properties of the APB shown in Figure 1c. The cyclic shifts only changed local atomic configurations at the APB interfaces. These changes are well visible on the order of different {001} planes containing both Fe and Al atoms (see the numbers 1–3 assigned to them). Their properties are given in Table 1.

Regarding APB energies, the atomic configuration in Figure 3a has it equal to 0.099 J/m${}^{2}$, i.e., very close to that which we obtained for the APB configuration in Figure 1c, 0.103 J/m${}^{2}$. In contrast, the APB energy of the configuration shown in Figure 3b is significantly lower, only 0.019 J/m${}^{2}$, while that of the configuration in Figure 3c is significantly higher, 0.165 J/m${}^{2}$. In order to explain the above discussed changes, we suggest to focus on differences in the Al concentration in the two adjacent {001} Fe-Al planes above and below the APB interface. In particular, the two Fe-Al planes above and below the APB in the middle of the supercell in Figure 1c contain 5 + 7 = 12 Al atoms (out of 18 atoms in total, i.e., 66.7 at.% Al). This is similar to 4 + 7 = 11 Al atoms (61.1 at.%) in the case of atomic configuration in Figure 3a. Both of these values are close to the average 60 at.% Al concentration in the Fe-Al planes in the B2 phase. In contrast, the Al concentration is significantly lower (4 + 4 = 8 Al atoms, 44.4 at.%) in the case of configuration visualized in Figure 3b and higher (7 + 7 = 14 Al atoms, 77.8 at.%) in Figure 3c. They represent models for fluctuations in the Al concentration at the APB interfaces.

The APB energies which increase with increasing average concentration of Al atoms in the two Fe-Al atomic planes adjacent to the APB interface can be approximated by a linear trend. The two quantities are thus correlated. The linear fitting function (for APB energies in J/m${}^{2}$) has the form $\langle \gamma^{\mathrm{APB}}\rangle =0.0043c^{\mathrm{Al}}-0.1695$, where $c^{\mathrm{Al}}$ is the concentration of Al in at.%. The level of correlation could be judged from the value of the $R^{2}$, which is equal to 0.9789. Regarding the other pair of {001} planes adjacent to APBs, which are formed by Fe-only planes, they are the same in all configurations.

**Figure 4:**
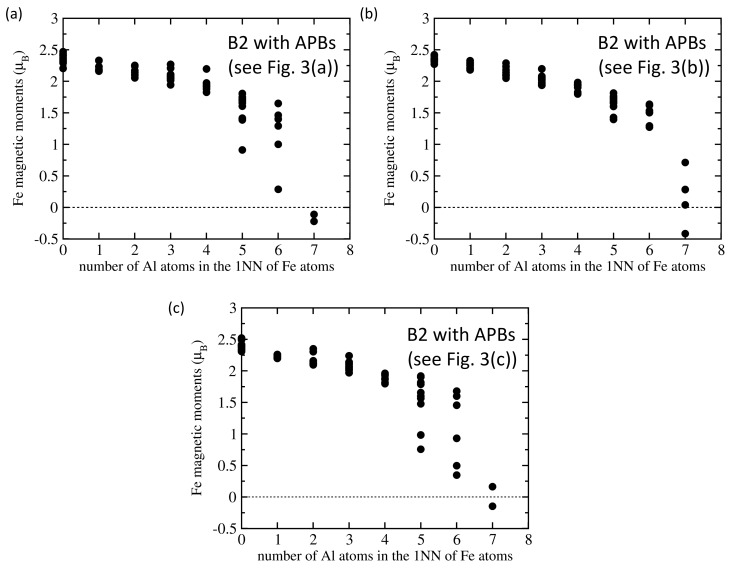
Computed local magnetic moments of Fe atoms in the supercells visualized in Figure 3a–c are summarized in parts (**a**–**c**), respectively, as a function of the number of Al atoms in the 1NN shell of Fe atoms.

As far as magnetic properties are concerned, the magnitude of local magnetic moments corresponding to the atomic configurations shown in Figure 3a–c are visualized in Figure 3d–f, respectively, by the diameter of spheres representing the atoms. The magnitudes of these moments are also summarized as functions of the number of Al atoms in the 1NN shells of Fe atoms in Figure 4. The obtained computed trends confirm the decrease of local magnetic moments of Fe atoms with the increasing number of Al atoms in the 1NN shell.

Statistical information for each configuration is given in Table 1. The configurations in Figure 3 have higher percentages of Fe atoms with lower number of Al atoms (1–4 Al atoms) in the 1NN shell and lower percentages of Fe atoms with 5 and more Al atoms in the 1NN shell (compared with the APB-free B2 phase). The increase of magnetism is smaller because some of the Fe atoms with higher number of Al atoms in the 1NN shell have their moments antiparallel to the moments of other Fe atoms. There is thus an APB-induced change from a ferromagnetic state of the B2 phase of Fe${}_{70}$Al${}_{30}$ to a ferrimagnetic one.

### 3.2. Calculations of Thermally-Induced APBs

**Figure 5:**
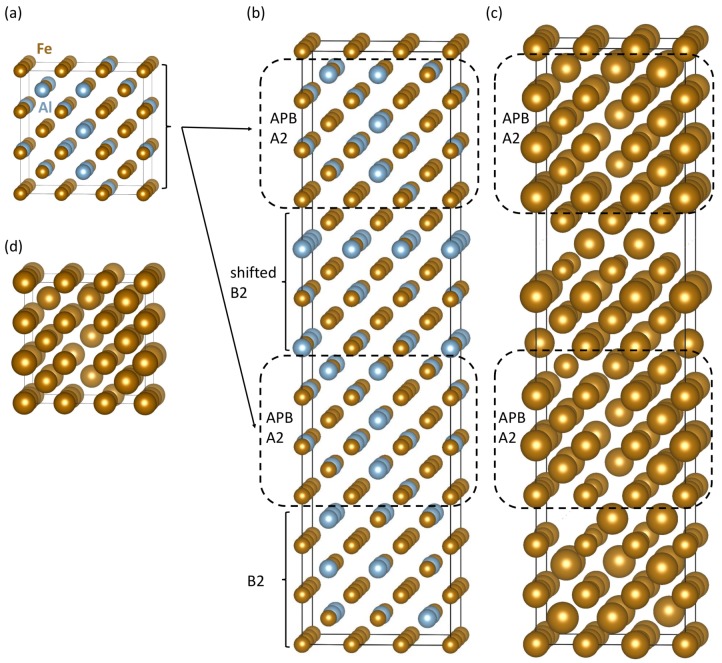
Schematic visualizations of thermal APBs in Fe${}_{70}$Al${}_{30}$ alloy. A 54-atom supercell in part (**a**) is a 3 × 3 × 3 multiple of 2-atom body-centered cubic (bcc) elementary cell and represents our model for a perfectly disordered A2 phase of Fe${}_{70}$Al${}_{30}$. It consists of atomic planes containing both Fe and Al atoms distributed according to the SQS concept. Two of these 54-atom A2-phase supercells are introduced at each of the two sharp APBs in the supercell shown in Figure 1b to form the calculated thermal APBs. The computed local magnetic moments of atoms in supercells (**a**,**b**) are shown in parts (**c**,**d**), respectively. The magnitude of local magnetic moments are visualized by the diameter of spheres representing atoms—the scaling is the same as in Figure 1 and Figure 3.

After studying APBs with sharp interfaces, we make an attempt to simulate thermally-induced ones which were experimentally probed by Murakami et al. [50]. In order to do so, we introduce an interlayer of disordered A2 phase to each of the two APB interfaces visualized in Figure 1c. We performed our simulations for this particular APB atomic configuration because it has a moderate value of the APB energy (0.103 J/m${}^{2}$) close to the average of the extreme values obtained for APB atomic configurations shown in Figure 3b,c. We model a disordered A2 phase by a 54-atom supercell visualized in Figure 5a where the atomic positions are generated according to the SQS concept.

There are six {001} atomic planes in our A2-phase supercell and its size in the 〈001〉 direction is about 0.9 nm. While it is less than the reported experimental values (2–3 nm), we consider properties of the A2 phase computed separately as a bulk as our model for the experimental thick layers. If we take the bulk APB-free B2 phase and bulk A2 phase as references and handle the atomic configuration in Figure 5b as a nanocomposite with four interfaces between the two phases (B2 and A2), the averaged interface energy 〈γ〉 is:

$$\langle \gamma \rangle =\left\{E_{216}\left(B2/A2/B2/A2\right)-\left(2\times E_{54}\left(B2\right)\right)-\left(2\times E_{54}\left(A2\right)\right)\right\}/\left(4\times A\right),$$

where $E_{216}\left(B2/A2/B2/A2\right)$ is the energy of the atomic configuration in Figure 5b, $E_{54}\left(B2\right)$ is the energy of the supercell in Figure 1a, $E_{54}\left(A2\right)$ is the energy of the supercell in Figure 5a and *A* is the interface area.

The calculated value equals to 0.083 J/m${}^{2}$. This averaged interface energy is listed in Table 2 and, importantly, it is lower than that of the sharp APB in Figure 1c. This indicates that the A2 layers would form if permitted by diffusional processes in the case of thermally-induced APBs.

**Figure 6:**
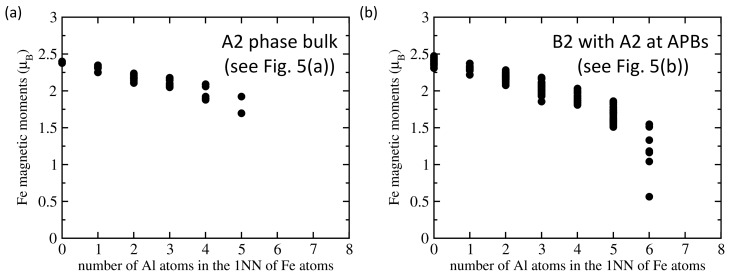
Calculated local magnetic moments of Fe atoms in the supercells visualized in Figure 5a,b as a function of the number of Al atoms in the 1NN. The local magnetic moments of Fe atoms in atomic configurations shown in Figure 5a,b are displayed in parts (**a**,**b**), respectively.

Regarding magnetic properties of the atomic configuration shown in Figure 5b, the magnitudes of local magnetic moments are visualized by the diameter of the spheres representing the atoms in Figure 5c. Furthermore, similarly as above, we also analyze the relation between the magnitude of local magnetic moments of Fe atoms and the number of Al atoms in their 1NN shell. The trends for the A2 phase (Figure 5a) and the thermally-induced APB (Figure 5c) are summarized in Figure 6a,b, respectively.

Comparing the plots shown in Figure 6a, which is related to the A2 phase, with that in Figure 6b with local magnetic moments of Fe atoms obtained for the thermally-induced APB, it is obvious that the disordered A2 phase has a higher number of Fe atoms with fewer Al atoms in the 1NN sphere. The percentages are listed in Table 2. While only 5% of Fe atoms have no Al atom in the 1NN shell, nearly one half (45%) of Fe atoms in the A2 phase has only 2 Al atoms as the first nearest neighbors and no iron atom exhibits more than 5 Al atoms in the 1NN shell. As all analyzed trends between the local magnetic moment of Fe atoms and the number of Al atoms in their 1NN shell show decreasing magnetic moments with increasing concentration of Al (see Figure 2, Figure 4 and Figure 6), the above discussed percentages found in the A2 phase mean that the Fe atoms in this phase would be more magnetically polarized than those in the B2 phase. A higher averaged magnetic moment of Fe atoms in the A2 phase (see Table 2) illustrates these findings.

## 4. Discussion

Our theoretical results qualitatively confirm the interpretation of experimental findings published by Murakami et al. [50] that the ferromagnetic state of APBs is stabilized by structural disorder within APBs. In particular, the thermally-induced disordered A2 phase at the APB interfaces contains Fe atoms with higher magnetic moments when compared with those in the B2 phase and, importantly, the A2 phase is in a ferromagnetic state. Both of these aspects are in agreement with experiments. However, our theoretical study provides also an atomic-scale type of information not available in the study [50].

Our results allow for obtaining a deeper insight into the actual mechanisms behind the observed phenomena and a better understanding of them. In particular, we clearly show that the local magnetic moments of Fe atoms decrease with an increasing concentration of Al atoms in the 1NN shell of the Fe atoms. This trend is in the case of the B2 phase clearly nonlinear when considering the whole concentration range of Al atoms (see Figure 2a). The decrease is weaker and quite linear for Fe atoms with up to 4 Al atoms in the 1NN shell (i.e., up to 50% of the atoms) but becomes much steeper for higher concentrations of Al atoms (Fe atoms with 7 Al atoms in the 1NN shell are nearly nonmagnetic—see Figure 2a). This nonlinear dependence of the magnetic moment of Fe atoms as function of the concentration of Al atoms in their 1NN shell is crucial for our understanding of differences between the B2 and A2 phases of Fe${}_{70}$Al${}_{30}$ alloy as discussed below.

Analyzing the structure of the B2 phase first, one half of its {001} atomic planes contains only Fe atoms and, consequently, all Al atoms are located in the other half of {001} atomic planes where the concentration of Al becomes much higher (60%) than the overall value of 30 at.% in the Fe${}_{70}$Al${}_{30}$. As the Fe-only and Fe-Al planes alternate in the B2 phase along the 〈001〉 direction, all those Fe atoms from the Fe-only atomic planes have the 1NN shell formed by atoms from the Fe-Al planes (and vice versa). Due to the fact that the average Al concentration in the Fe-Al planes is 60%, the Fe atoms from the Fe-only planes have their magnetic moments significantly reduced. Two aspects are important for the overall reduction of the magnetization. First, the reduction of the local magnetic moments is more significant because the above discussed stronger decrease has onset for the Al concentration equal to ≈50 at.%. Second, the Fe atoms from the Fe-only planes, which have their magnetic moments nonlinearly reduced, represent 5/7 of all Fe atoms. The remaining 2/7 of Fe atoms in the Fe-Al planes have their 1NN shell full of Fe atoms (from the Fe-only planes) and their magnetic moment reaches the maximum values, but they represent only minority of all Fe atoms.

The situation in the A2 phase is quantitatively very different. All of the atomic planes have on average the same Al concentration and it is only 30 at.%. Leaving aside local fluctuations, 30% is then also the average concentration of Al atoms in the 1NN shells of all Fe atoms. Considering the fact that the decrease of the magnetic moment is weaker for concentration of Al atoms below 50% (prior the onset of nonlinearly stronger reduction), the magnetic moments of Fe atoms in the A2 phase will be reduced less (see Figure 6a) than those in the B2 phase (see Figure 2a).

### 4.1. Linear Relation between the Al Concentration and the Energy of Sharp APBs

Another insight obtained from our simulations of sharp APBs in the B2 phase is the theoretically identified relation between the average APB energy and the concentration of Al atoms in the two atomic planes adjacent to the APB interface. The simulated APB shift leads to the situation when these planes are formed by either two Fe-only planes or two Fe-Al planes (each containing both Fe and Al atoms). None of these APB-related atomic environments exists in the APB-free B2 phase. We have performed calculations of four different sharp APBs in the B2 phase (Figure 1c and Figure 3a–c) which all contained one APB interface formed by two Fe-only planes (identical in all four atomic distributions) but differ in the concentration of Al in the pair of APB-adjacent Fe-Al planes. The averaged APB energy turns out to decrease with a decreasing concentration of Al atoms in these two Fe-Al planes (within the range of Al concentrations between 8/18, i.e., 44.4%, and 14/18, i.e., 77.8%). Despite the fact that this relation is deduced from only a few computed cases and concentrations of Al, it can help us to explain the formation of the A2 phase at APBs. In particular, sharp APBs in the B2 phase with two interfacing Fe-Al {001} planes would have the average concentration of Al atoms close to that in these planes, i.e., 60 at.% Al. When an A2 phase forms at the APB interface and separates the pair of Fe-Al planes of the B2 phase by atomic planes of the A2 phase, the concentration of Al in the pair of planes adjacent to the newly formed two interfaces is lower (only (60 + 30)/2 = 45%) because the average concentration of Al in the A2 atomic planes is on average only 30%. According to the above discussed relation between the average APB energy and the Al concentration, the energy of the newly formed B2/A2 interfaces would be lower than the original sharp APBs in the B2 phase.

### 4.2. Thermodynamic Stability of the APB Interface States

The change of the Al concentration at the APB interfaces by the formation of the A2 phase to 45 at.% deserves further attention as it can be partly justified in the context of thermodynamic stability of the binary Fe-Al system. According to the thermodynamic assessment by Sundman and co-workers [65], the compositional dependence of the enthalpy of formation has the minimum close to 50 at.% of Al in a phase, which is close to ordered stoichiometric FeAl with the CsCl structure. The experimental data also show that the crystallographic structures for Al-rich compositions do not have atoms in positions related to a bcc lattice. The pair of adjacent disordered Fe-Al planes with the Al concentrations on average equal to 60 at.% is therefore very likely to have a high energy. The reasons are related to the differences from the stoichiometric ordered FeAl phase in the minimum of the enthalphy curve: the Al concentration is much too high, the atoms are disordered, and the structure is not the one minimizing the enthalpy for this Al concentration. The above discussed reduction of the Al concentration of the two APB-adjacent Fe-Al layers from 60 at.% to 45 at.% (A2/B2 interface) changes the Al concentration closer to the 50 at.% for which the enthalpy has the minimum.

The thermodynamic perspective can help us to explain why the insertion of A2 phase between the two Fe-only atomic planes of the originally sharp APB in the B2 phase of Fe${}_{70}$Al${}_{30}$ would result in a decrease of the energy. The two Fe-only adjacent planes form an environment that is similar to that in the elemental ferromagnetic bcc Fe. However, the elemental Fe is, from the thermodynamic point of view, not preferred over Fe-Al states with less than 50 at.% Al (the above discussed enthalpy minimum is found for the FeAl compound [65]). Therefore, the energy can be expected to decrease due to locally increasing Al concentration. This happens exactly at the sharp APB interface formed by two Fe-only planes when one of them is replaced by an atomic plane of the A2 phase containing on average 30 at.% of Al. The average Al concentration of the pair formed by one Fe-only plane and one A2-phase plane would be 15 at.%. In the equilibrium phase diagram, this concentration corresponds to a disordered solid solution of Al atoms in a bcc Fe matrix. Therefore, a local atomic distribution in the pair of those two APB-related adjacent planes would be quite similar to the equilibrium one.

### 4.3. A Comparison of Thermodynamic Stability of the B2 and A2 Phase

However, the studied systems are not formed only by the two atomic planes adjacent to the APB interface. Regarding the formation of the A2 phase at the APBs, it should be noted that, according to our calculations, the energy of the A2 phase of Fe${}_{70}$Al${}_{30}$ is by 18.5 meV per atom higher than that of the B2 phase. Therefore, the above described process which reduces the APB energy of sharp APBs by formation of the A2 phase is, in fact, a complex competition among several different mechanisms. The energy of the atomic planes at the APBs is, on one hand, reduced by changing from sharp APBs in the B2 phase to energetically less costly B2/A2 interfaces, but, on the other hand, the number of the A2/B2 interfaces is twice as high, and the A2 phase itself has a higher energy. Another fact, which can be important at elevated temperatures, is that the configurational entropy of the A2 phase is different from that of the B2 phase. We therefore evaluate the ideal molar configurational entropy $S^{\mathrm{conf}}$ below.

As the B2 and A2 phases exhibit different numbers of ordered and disordered atomic sites (sublattices), we use a generalized formula (see, e.g., Ref. [66]) derived for the sublattice model [67] $S^{\mathrm{conf}}=-R\sum_{\alpha }a_{\alpha }\sum_{i}f_{i}^{\alpha }\ln f_{i}^{\alpha }$ where *R* is the universal gas constant, *i* runs over different chemical species, α over different sublattices, $a_{\alpha }$ is the ratio of lattice sites of a sublattice α with respect to the total number of all lattice sites, and $f_{i}^{\alpha }$ is the concentration of a chemical species *i* on a sublattice α. The B2 phase has only one half of planes disordered, and the Al concentration in these disordered planes equals 60 at.%. The A2 phase has all lattice sites fully disordered, and the Al concentration is equal to 30 at.%. The molar configurational entropy (in the units of *R*) of the B2 phase amounts to 0.3365 and that of the A2 phase is equal to 0.6109. If the energy difference of 18.5 meV per atom is to be compensated solely by the difference in the configurational entropy, it would happen at the temperature of 784 K. The experimental B2–A2 second-order transition temperature is significantly higher, 1287 K [68], but there are several good reasons for this discrepancy. First, our calculations for static lattices did not include any phonons or magnons and, second, more importantly, the computed energy difference is between two ferromagnetic states while the experimental transition occurs above the Curie temperature between two paramagnetic states. The two above-mentioned temperatures cannot be, therefore, directly compared. It is worth noting that the above discussed competition of different phenomena would likely limit the width of interlayers formed at the APBs by the A2 phase and make the width of A2 layers rather sensitive to the temperature as well as to other conditions, such as a thermo-mechanical history of the samples.

### 4.4. Magnetism of Both Sharp and Thermally-Induced APBs

The existence of the thermally-induced A2 phase at the APBs is really crucial for the increase of magnetism detected in experiments by Murakami et al. [50]. The sharp APBs in the B2 phase do not often increase the magnetization enough (see the average magnetic moments of Fe atoms in Table 1). This is due to the fact that three of the four computed atomic configurations of sharp APBs induce a transition from a ferromagnetic state to a ferrimagnetic one and the magnetic moments with antiparallel orientation reduce the total magnetic moment. We expect that these ferro-to-ferrimagnetic transitions would be rather common close to the sharp APBs in the B2 phase because they are induced by APBs with the APB energies from the whole range of computed values (see them in Table 1 for the atomic configurations shown in Figure 3). Despite the fact that the antiparallel orientation is obtained only in the case of one or two atoms (out of 76 Fe atoms in our 108-atom supercells) and the magnitudes of these antiparallel magnetic moments are rather small (under 0.5 $\mu_{B}$), the phenomenon can be possibly enhanced by temperature effects or other conditions. In fact, all four studied sharp APBs in the B2 phase exhibit slightly higher values of the average magnetic moment of Fe atoms than the APB-free B2 phase (in particular by 9.3% in the case of the atomic configuration in Figure 1c). However, it is the inception of the A2 phase at the APB interface which increases the average magnetic moment further more. The whole B2/A2 nanocomposite in Figure 5b shows a 11.5% higher averaged magnetic moment of Fe atoms than that in the APB-free B2 phase. This increase is still moderate, but we should keep in mind the fact that the A2 phase layers at the APBs in experiments are much thicker (2–3 nm) than our simulated ones (about 0.9 nm in Figure 5b). Such a thick A2 phase would have magnetic properties similar to those which we obtained for the bulk A2 phase (see Figure 5a,d). The average magnetic moment in the A2 phase would then be significantly higher, by 17.5%, than that in the APB-free bulk B2 phase.

The increase of the averaged magnetic moment of Fe atoms in the A2 phase by 17.5% (w.r.t to the APB-free B2 phase of Fe${}_{70}$Al${}_{30}$) is still not directly comparable with the experimental increase by 60% reported by Murakami et al. [50]. When searching for reasons for this discrepancy, it is worth mentioning that Murakami et al. detected the magnetic flux density at the APBs at 293 K while our quantum-mechanical study performed for static lattices (corresponding to very low temperatures close to 0 K) was focused on changes in the magnetic moments of individual atoms. The experimental change of the magnetic flux density is thus not directly comparable with the theoretical increase of the average magnetic moment of Fe atoms. However, our study provides a very valuable insight into thestructure–property relations connecting (i) the local atomic (dis)order and details of atomic configurations (including chemical composition) on one hand and (ii) the values of local magnetic moments of individual Fe atoms on the other hand. We therefore hope that the above identified and analyzed mechanisms, which increase the average magnetic moments of Fe atoms, are among the decisive ones when interpreting the experimental data reported by Murakami et al. [50].

Finally, the identified mechanism of increasing the magnetic properties in materials by introducing thermally-induced APBs with disordered phases can possibly be used as a designing principle when developing new magnetic materials. It should be applicable when magnetic species co-exist with some other (non-magnetic) chemical species which decrease the magnetic moment of the magnetic elements. If this reduction of magnetism is enhanced by thermodynamically-driven formation of ordered sublattices, then the APBs offer a way of decreasing the level of order in the system and that results in a statistically higher probability of magnetic species to be in magnetically more favorable environment (see, e.g., our recent study of impact of APBs in Fe-Al-Ti [69]).

## 5. Conclusions

We have performed an ab initio study of B2 phase of Fe${}_{70}$Al${}_{30}$ alloy with and without antiphase boundaries (APBs). Our study was motivated by experimental findings by Murakami et al. [50] who reported higher magnetic flux density from A2-phase interlayers at the thermally-induced APBs in Fe${}_{70}$Al${}_{30}$. They suggested to connect the enhancement of the ferromagnetism with the disorder in the A2 phase. We show that the averaged magnetic moment of Fe atoms in the A2 phase is by 17.5% higher than that in the B2 phase. While we can not treat the A2 layers of the experimental thickness (2–3 nm [50]), our simulations of thinner (about 0.9 nm) A2 layers within a B2/A2 nanocomposite resulted in the average magnetic moment of Fe atoms by 11.5% higher than that in the APB-free B2 bulk. We explain the changes in magnetism by (dis)order-dependent reduction of local magnetic moments of Fe atoms by Al atoms in the 1NN shell of Fe atoms (see also Refs. [22,39]). This effect is synergically combined with the influence of APBs, which provide local atomic configurations not existing in a APB-free bulk. The studied sharp APBs can increase the local magnetic moments of Fe atoms, but they more often lead to an APB-induced ferromagnetic-to-ferrimagnetic transition.

Regarding the formation of the A2 phase at the APBs, we link it to the energetics of atomic configurations occurring at both the sharp and A2-containing thermal APBs in the B2 phase of Fe${}_{70}$Al${}_{30}$. The studied sharp APBs have rather low APB energies (between 0.019 and 0.165 J/m${}^{2}$), and these were found to be increasing with increasing Al concentration in the two atomic planes adjacent to the APB interface. These two atomic planes represent local atomic configurations which are APB-specific and have either much too high or much too low concentration of Al. The insertion of A2-phase atomic planes leads to the change of Al concentration accompanied by lowering of the energy. This mechanism can be understood in terms of equilibrium thermodynamic of the Fe-Al binary system (the enthalpy has the minimum for the Al concentration close to 50 at.%). The studied mechanism of increasing the magnetic properties by introducing thermally-induced APBs with disordered phases can possibly be used as a designing principle when developing new magnetic materials.

## Figures and Tables

**Table 1:** Computed properties of the studied atomic configurations including the B2 phase without APBs as well as variants of APB-containing B2 phase. The table summarizes volumes, APB energies in the case of APB-containing configurations, percentages of Fe atoms with different number of Al atoms (from 0 to 8) in the 1NN shell and the average value of magnetic moments of Fe atoms $\langle \mu^{\mathrm{Fe}}\rangle$.

	Volume	$\langle \gamma^{\mathrm{APB}}\rangle$	% of Fe Atoms with # Al Atoms in 1NN									$\langle \mu^{\mathrm{Fe}}\rangle$
	**(Å${}^{3}$/atom)**	**(J/m${}^{2}$)**	0	1	2	3	4	5	6	7	8	**($\mu_{B}$)**
			Al	Al	Al	Al	Al	Al	Al	Al	Al	
B2 phase—Figure 1a	11.80	–	29	0	3	11	13	26	13	5	0	1.83
B2 with APB—Figure 1c	11.98	0.103	20	8	12	20	9	20	9	3	0	2.00
B2 with APB—Figure 3a	11.89	0.099	20	8	12	20	9	20	9	3	0	1.86
B2 with APB—Figure 3b	11.92	0.019	16	11	11	20	12	17	9	5	0	1.85
B2 with APB—Figure 3c	11.98	0.165	24	8	13	18	11	16	8	3	0	1.89

**Table 2:** Calculated properties of the studied atomic configurations of the A2 phase as a bulk and at the APB interface as a model for the thermally-induced APBs. The table summarizes volumes, APB energies, percentages of Fe atoms with different number of Al atoms (from 0 to 8) in the 1NN shell of the Fe atoms and the average value of magnetic moments Fe atoms $\langle \mu^{\mathrm{Fe}}\rangle$.

	Volume	$\langle \gamma^{\mathrm{APB}}\rangle$	% of Fe Atoms with # Al Atoms in 1NN									$\langle \mu^{\mathrm{Fe}}\rangle$
	**(Å${}^{3}$/atom)**	**(J/m${}^{2}$)**	0	1	2	3	4	5	6	7	8	**($\mu_{B}$)**
			Al	Al	Al	Al	Al	Al	Al	Al	Al	
A2 phase	12.14	–	5	13	45	18	13	5	0	0	0	2.15
B2 APB with A2	12.02	0.083	15	10	23	15	17	14	5	0	0	2.04

**Figure A1:**
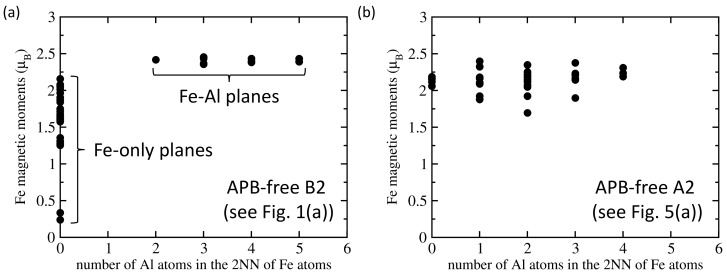
Calculated local magnetic moments of Fe atoms as a function of the number of Al atoms in their second nearest neighbor (2NN) shell of the Fe atoms. Part (**a**) summarizes them in the APB-free bulk B2 phase (visualized in Figure 1a) and part (**b**) for the APB-free bulk A2 phase shown in Figure 5a.

## Appendix A

Figure A1 shows computed dependences of local magnetic moments of Fe atoms as functions of the number of Al atoms in the second nearest neighbor shell (2NN) of the Fe atoms for the APB-free bulk B2 phase (see Figure A1a) and the APB-free bulk A2 phase (see Figure A1b). As far as the values for the former are concerned, the Fe atoms located in the Fe-only planes in the B2 phase have their second nearest neighbors formed by the other Fe atoms on this sublattice. As it is Fe-only sublattice, they have no Al atoms in the 2NN shell and their magnetic moments (which vary significantly) seem to be determined by atoms in other shells, apparently by those in their 1NN shell (see Figure 2a). The Fe atoms on the mixed Fe-Al sublattice in the B2 bulk phase have their 2NN atoms similarly represented by other atoms on this mixed sublattice and can have some Al atoms in their 2NN shell. However, the computed values show very weak dependence on the number of 2NN Al atoms (see Figure A1a). Regarding the bulk A2 phase, there is only one type of mixed Fe-Al sites, but, again, the calculated values of local magnetic moments of the Fe atoms seem to depend on the number of Al atoms in the 2NN shell of the Fe atoms only very weakly (see Figure A1b). A weaker impact of the second coordination sphere found in this study is in line with our recent results related to the Fe-Al system [39,41].
